# A comparative assessment of two tools designed to support patient safety culture in UK general practice

**DOI:** 10.1186/s12875-021-01438-4

**Published:** 2021-05-21

**Authors:** Ian Litchfield, Kate Marsden, Lucy Doos, Katherine Perryman, Anthony Avery, Sheila Greenfield

**Affiliations:** 1 Institute of Applied Health Research, College of Medical and Dental Sciences, University of Birmingham, Birmingham, UK; 2 Division of Primary Care, School of Medicine, University of Nottingham, Nottingham, UK; 3 Institute of Cancer and Genomic Sciences, College of Medical and Dental Sciences, University of Birmingham, Birmingham, UK; 4 Alliance Manchester Business School, University of Manchester, Manchester, UK

**Keywords:** General practice, Patient safety, Safety culture, Health service delivery

## Abstract

**Background:**

The NHS has recognised the importance of a high quality patient safety culture in the delivery of primary health care in the rapidly evolving environment of general practice. Two tools, PC-SafeQuest and MapSaf, were developed with the intention of assessing and improving patient safety culture in this setting. Both have been made widely available through their inclusion in the Royal College of General Practitioners’ Patient Safety Toolkit and our work offerss a timely exploration of the tools to inform practice staff as to how each might be usefully applied and in which circumstances. Here we present a comparative analysis of their content, and describe the perspectives of staff on their design, outputs and the feasibility of their sustained use.

**Methods:**

We have used a content analysis to provide the context for the qualitative study of staff experiences of using the tools at a representative range of practices recruited from across the Midlands (UK). Data was collected through moderated focus groups using an identical topic guide.

**Results:**

A total of nine practices used the PC-SafeQuest tool and four the MapSaf tool. A total of 159 staff completed the PC-SafeQuest tool 52 of whom took part in the subsequent focus group discussions, and 25 staff completed the MapSaf tool all of whom contributed to the focus group discussions. PC-SafeQuest was perceived as quick and easy to use with direct questions pertinent to the work of GP practices providing useful quantitative insight into important areas of safety culture. Though MaPSaF was more logistically challenging, it created a forum for synchronous cross- practice discussions raising awareness of perceptions of safety culture across the practice team.

**Conclusions:**

Both tools were able to promote reflective and reflexive practice either in individual staff members or across the broader practice team and the oversight they granted provided useful direction for senior staff looking to improve patient safety. Because PC SafeQuest can be easily disseminated and independently completed it is logistically suited to larger practice organisations, whereas the MapSaf tool lends itself to smaller practices where assembling staff in a single workshop is more readily achieved.

## Introduction

The concept of organisational safety culture first emerged from the nuclear industry in the mid-1980s and is defined by the level of commitment of an organisation and its employees toward “values, attitudes, and patterns of behaviour” to successfully manage and improve health and safety [1, 2]. Since then understanding and promoting safety culture has been accepted as a challenging yet critical process across a number of high risk industrial sectors including aviation mining [3–5] and more recently healthcare [6–8]. To be effective this culture requires the workforce share perceptions of the importance of safety and also engage in generating ideas that can reduce ‘risk, accidents and ill health’ [9–12]. In industry several approaches have been developed to examine and promote safety culture including checklists [13, 14] and competency assessments [15].

Over the last decade the United Kingdom’s (UK) National Health Service (NHS) has recognised the importance and challenges of developing a culture that prioritises and maximises the safety of the care it provides [16–19]. Facilitating this within such a diverse organisation is a difficult task, particularly so in primary care where a diverse range of general practices are expected to provide equitable and consistently safe care for their patients despite evolving and complex demands on their services [20, 21]. This has been highlighted by the recent pandemic [22–26] where significant changes in care provision were introduced without the recommended period of consultation, implementation, and evaluation [27, 28].

One way to mitigate the risks to patient safety incurred by the sudden introduction of new methods of working is by providing the means for the entire practice team to reflect on the care they provide [29–31]. Two tools have been made available through the Royal College of General Practitioners (RCGP) website as part of their patient safety toolkit [32, 33] that can readily fulfil this function [34, 35]. They both explore a number of key domains of patient safety culture but utilise two very different approaches with varying implications for the time and resource required for their completion and the way in which their outputs are described and presented; the NHS Education for Scotland’s Primary Care Safety Questionnaire (PC-SafeQuest) uses a confidential online questionnaire [36] and the Manchester Patient Safety Framework (MaPSaF) involves a moderated workshop attended by a range of practice staff [35].

The use of either tool is not mandated but implemented at the discretion of individual practices. The work we present here is positioned to help General Practitioners (GPs) and other senior staff make an informed judgement on which of these tools might best suit their organisation. To help support this decision we conducted a comparative content analysis of each tool which provides context for a qualitative exploration of the experiences of staff that had used both [37]. By combining these complementary methodological approaches our research intended to provide timely and valuable insight into how these tools might be most appropriately used and the practical benefits they offer in support of patient safety in primary care practices.

## Method

### Design

The study consisted of two phases; the first phase consisted of a comparative content analysis where both tools were assessed against ten previously identified criteria considered to be key in the effective assessment of patient safety culture [38, 39]; in the second phase both tools were introduced to the same general practice surgeries and the experiences of staff that used each tool were captured via separate moderated focus groups. Some staff would have taken part in two focus groups having used both tools. The data was analysed using recognised measures associated with successful implementation.

### Settings

A total of nine practices of varying characteristics were recruited from across three clinical commissioning groups (CCGs) within the central region of the UK; North Staffordshire, Moorlands and Shropshire, and Wolverhampton.

### Participants/recruitment

Practices were recruited as part of the broader Patient Safety Toolkit (PST) project [40] a process facilitated by the National Institute for Health Research (NIHR) Clinical Research Network (CRN) [41] who contacted every practice across all CCGs, describing the premise of the PST and inviting each to participate. Those practices that expressed an interest were visited by a member of the study team (KM, LD) to discuss the practicalities and benefits of their involvement and ultimately nine were purposively selected [42] to create a sample incorporating a range of list sizes, and socio-economic environments representative of the range of practices found in English primary care [40]. The sample size considered appropriate to provide practical insight into the use of each tool and inform the future development of the patient safety toolkit [40].

### The tools

#### PC-SafeQuest safety climate survey

PC-SafeQuest was designed and validated by NHS Education for Scotland to grant senior clinical and management staff an understanding of how their colleagues perceive and promote patient safety within their organisation [36]. It consists of on an online survey issued to each member of practice staff containing 30 questions presented within six safety-related domains where staff present their answer on a Likert scale [36]. The scores for each domain are compiled and a report generated for the practice which can be compared over time against previous practice scores or, via the PC-SafeQuest portal, with similar practices [36]. The key characteristics of PC-SafeQuest are summarised in Table 1.

**Table 1:** Key characteristics of PC-SafeQuest and MaPSaF patient safety tools

	**PC-SafeQuest**	**MaPSaF**
**Aim**	To survey patient safety climate and inform patient safety improvement.	To facilitate improvements in safety culture through constructive reflective practice.
**Facilitated**	Self-completed with a summary report automatically generated.	Led by an independent moderator.
**Level of anonymity**	Anonymous and completed confidentially.	Group members known to each other.
**Number of participants**	All staff in the practice.	Up to 12 members of practice staff.
**Staff groups involved**	All grades of staff	All grades of staff
**Format**	Online questionnaire	Workshop and group discussion
**Structure of the tool**	A total of 30 questions within 6 domains. Completed questionnaires are collated for each practice scores produced for each domain in a final report produced by PC-SafeQuest.	An evaluation sheet consisting of nine domains with the option of selecting one of 5 levels of ‘maturity’ for each. This is to be completed by each participant during the workshop and the results to be discussed as a group.
**Outputs**	Generation of report where scores can be compared with previous reports from that practice or practices of similar characteristics.	Discussion of evaluation sheet results as part of workshop identifies any areas that might need to be looked at.
**Time to complete**	10 min for online questionnaire. (The time taken by senior staff to assimilate and act on this data was not formally recorded).	60–120 min

#### Manchester Patient Safety Framework (MaPSaF)

The MaPSaF tool creates a forum for team-based reflection on multiple dimensions of patient safety [35]. First asking pairs of participants to evaluate their practice within one to five levels of organisational development across nine domains of safety culture, based on Westrum’s theory which classifies organisations dependent upon the degree of unity and motivation that staff share with their organisation’s over-arching goals [35, 43, 44]. The tool is facilitated by a trained moderator during a workshop which can last between 1 and 2 h and accommodate up to 12 staff [45]. The key characteristics of MapSaf are summarised in Table 1.

### Data collection

#### Phase one: content analysis

We accessed and analysed the content of both tools alongside the information supporting their use via the Patient Safety Toolkit (PST) portal accessed through Royal College of General Practitioner’s website [32, 36, 45].

#### Phase two: qualitative assessment

Focus groups were chosen to gather staff perspectives because of their ability to promote challenge and produce consensus [46] as opposed to isolating opinions specific to a certain job title or role [47], reflecting the aim of the tools to promote collective reflection. The decision to anonymise participant identity was made to elicit totally honest comments regarding the efficacy of the tools and specific examples of how they uncovered culture related safety incidents within individual practices [48]. We were also wary of the vicarious disclosure of participant identity that can occur in groups where individuals are already known to one another, which might have adversely impacted the working relationships within the practices involved and the intended arc of the PST project [49]. The MapSaf focus groups were convened immediately following the use of the tool, with the same participants. The PC-SafeQuest focus groups were convened between approximately 6 and 8 weeks after the tool was completed and results were returned to the practice.

Ethical approval was obtained from East Midlands—Nottingham 1 Research Ethics Committee – REC/REF—13/EM/0258 15 July 2013 for all organisations involved. All methods were performed in accordance with the relevant guidelines and regulations in line with this approval. All participants were over 18 and provided signed consent prior to the beginning of each focus group. The same semi-structured topic guide was used for MapSaf and PC-SafeQuest and contained questions on staff expectations of the tool, their experience of its application including its ease of use, and what if any action has or would be taken as a result. These questions are presented in Table 2. All focus group discussions were moderated by an experienced Research Fellow (LD) who strived to ensure that all voices were heard, digitally recorded, professionally transcribed and the data ultimately managed within NVivo 10.

**Table 2:** Questions for focus group

**How easy was the tool to use?**
• Were there any issues regards facilitating their use?
• Did individuals understand what was being asked?
• Were the demands on time and resource as you expected?
• Were there any significant/unforeseen issues?
**How effective or useful was the tool?**
• Which elements or domains did you find most useful for your practice?
• Which elements or domains did you find least useful for your practice?
• Were you surprised at the findings that emerged?
• What changes might you make as a result of using the tool?
**In terms of its future use…**
• How would you feel about the practice using it again at a later stage?
• Would you recommend other practices use it?

### Analysis

#### Phase one: content analysis

We used a comparative content analysis [50, 51] where both tools were interrogated for the presence of the ten key dimensions of an effective patient safety culture tool [38, 39]. In summary these were; 1) Leadership 2) Safe systems and processes 3) Resources, including training and equipment 4) Interpersonal relationships, including teamwork and collaboration 5) Communication, including raising issues with senior staff 6) Learning from mistakes 7) Characteristics of staff, including workload and stress 8) Awareness and priority of patient safety 9) Safety incentives and rewards and 10) Safety issues witnessed and reported.

#### Phase two: qualitative assessment

For the qualitative data a post-hoc deductive analysis [52] was conducted where transcripts of the group discussions were searched for text relating to three characteristics (acceptability, appropriateness, and feasibility) regarded as instrumental to the early stages of successful implementation and predictive of sustained adoption [37, 53] based on Roger’s theory of diffusion [37]. These are further defined alongside their theoretical basis in Table 3. In undertaking the analysis, each of IL, KM, KP, and SG independently reviewed a sample of the transcripts for data relating to the three characteristics and decisions regarding where text should be placed were discussed and consensually decided. The remainder of the analysis was undertaken by IL with the final interpretation agreed by all authors.

**Table 3:** Key implementation outcomes, their definition and theoretical basis

**Implementation outcome**	**Definition**	**Theoretical basis**
**Acceptability**	Satisfaction with various aspects of the innovation (e.g. content, complexity, comfort, delivery, and credibility).	Concerning the complexity and relative advantage of the intervention where “Complexity” is a measure of the degree to which an innovation is perceived as difficult to understand and use [54] and relative advantage is The degree to which an innovation is perceived as better than the idea it supersedes.
**Appropriateness**	Perceived fit; relevance; compatibility; suitability; usefulness; practicability	A measure of the degree to which an innovation is perceived as being compatible with existing values, past experiences, and the needs of potential adopters [54]
**Feasibility**	Actual fit or utility; suitability for everyday use; including the ease with which it can be piloted or trialled.	Alongside the concept of compatibility, feasibility also includes Roger’s concept of trialability i.e. the degree to which the innovation may be piloted and modified [54].

## Results

### Phase 1: content analysis

Table 4 illustrates the similarities and differences between the content of the two tools in comparison with the ten criteria identified from the most recent and comprehensive review of tools of safety culture assessment [38, 39]. Out of the ten criteria, nine were covered by at least one of the tools with the exception of “Other means of prioritising safety (such as through rewards and incentives)” i.e. the presence of or adherence to initiatives intended to improve or promote patient safety which neither tool addressed. Both tools explored “The quality of interpersonal relationships (such as teamwork, collaboration within and across units)” and “Communication, particularly about safety, including perceptions of being able to report and speak up” describing the ability to communicate about safety and freely raise patient safety concerns. Areas of difference were that PC-SafeQuest specifically asked questions regarding “Leadership, particularly the support of safe practice” and the “Systems, procedures and processes exist that normalise or enshrine patient safety, or which are adhered to” and MapSaf explored the degree to which “Resources for safety (such as staffing, equipment, training)” were made available for patient safety, the “focus on learning from mistakes, responding and improving systems”, and the investigations of “Actual safety issues witnessed reported”.

**Table 4:** Key dimensions of patient safety culture and the related domains in PC-SafeQuest and MapSaf

**Dimensions of safety culture**	***PC-SafeQuest***		***MapSaf***	
	*Related domain*	*Specific questions (closed)*	*Related domain*	*Specific questions (open)*
**1. Leadership, particularly the support of safe practice**	*Leadership*	Is the hierarchy in the practice a barrier to effective working? Will highlighting a significant event likely result in negative repercussions for the person raising it? Does the practice leadership deal effectively with problem team members? How seriously do senior staff take suggestions that might improve how things are done? Is there a low level of trust between staff members? How frequently do staff disregard rules, protocols and procedures?	***Not covered***	
**2. Systems, procedures and processes exist that normalise or enshrine patient safety, or which are adhered to**	Safety Systems	Are all staff encouraged to highlight significant events? Do practice procedures help to prevent significant events from happening? Does the development of practice protocols use inputs from all staff? Does the practice take the time to formally assess risks (e.g., to patients, colleagues, and to the practice)? Do all staff have the opportunity to participate in the analysis of significant events? Do you think the quality and safety of patient care in your practice is taken seriously?	***Not covered***	
**3. Resources for safety (such as staffing, equipment, training)**	***Not covered***		Staff education and training about safety issues	How, why and when are education and training programmes about patient safety developed? What do staff think of them?
**4. The quality of interpersonal relationships (such as teamwork, collaboration within and across units)**	Team working	Do all staff treat each other with respect? Do all staff always support one another? Are disagreements amongst staff resolved appropriately? Do staff at all levels within the practice work well together? Is your practice a good place to work? Are staff generally satisfied with their jobs? Is the need to work well as a team promoted by the practice leadership?	Team working around safety issues	How and why are teams developed? How are teams managed? How much team working is there around patient safety issues?
**5. Communication, particularly about safety, including perceptions of being able to report and speak up**	Communication	Do all staff at your practice feel free to question the decisions of those with more authority? Are all staff comfortable in expressing concerns to the practice leadership about how things are done in the practice? Is there open communication between colleagues across all levels? Are all staff kept up to date about practice developments? How effectively does the practice leadership communicate its vision for the development of the practice?	Communication about safety issues	What communication systems are in place? What are their features? What is the quality of record keeping to communicate about safety like?
**6. A focus on learning from mistakes, responding and improving systems**	***Not covered***		Perceptions of the causes of PSIs and their identification	What sort of reporting systems are there? How are reports of incidents received? How are incidents viewed, as an opportunity to blame or improve?
			Investigating PSI incidents	Who investigates incidents and how are they investigated? What is the aim of the organisation? Does the organisation learn from the event?
**7. Individual staff characteristics and perceptions of their effect on work (such as job satisfaction, stress)**	Workload	Is the performance of staff impaired by excessive workload? Do all staff have enough time to complete tasks safely? Is the level of staffing in the practice sufficient to manage the workload safely? When pressure builds are staff expected to work faster even if it means taking shortcuts?	***Not covered***	
**8. General awareness of patient safety and/or it being a priority**	***Not covered***		Priority given to patient safety	How seriously is the issue of patient safety taken within the organisation? Where does responsibility lie for patient safety issues?
**9. Other means of prioritising safety (such as through rewards and incentives)**	***Not covered***			
**10. Actual safety issues witnessed reported**	***Not covered***		Investigating patient safety issues	Who investigates incidents and how are they investigated? What is the aim of the organisation? Does the organisation learn from the event?

### Phase II: qualitative assessment

#### Practice characteristics

Of the nine practices that were recruited patient list sizes ranged from 919 to 12,246 patients and the number of GPs from three to seven. One was a teaching practice and two were dispensing practices and the characteristics of all participating practices are summarised in Table 5. All nine practices totalling 159 staff completed the PC-SafeQuest tool, four practices used the MaPSaF as after recruitment five practices reported their inability to incorporate an additional training session within the time-frame of the study. A total of thirteen focus groups were convened; nine discussed the PC-SafeQuest tool and four the MapSaf tool involving 54 and 25 participants respectively and they lasted between 14 and 35 min. Participants included GPs, nurses, and administrators and the job titles of participants within each focus group is summarised in Table 6.

**Table 5:** Characteristics of participating practices

**Practice ID**	**Tool Used**	**Number of patients**	**Number of GPs**	**Number of nurses**	**Number of healthcare assistants**	**Number of admin. staff**	**Number of managerial staff**	**Deprivation score**^**c**^	**Quality Outcomes Framework**^**d**^	**Clinical Commissioning Group**
01^a^	PC-SafeQuest MapSaF	9390	7	2	3	18	1	9	988	Staffordshire Moorlands and Shropshire CCG
02	PC-SafeQuest	6577	3	3	2	11	1	3	993	Staffordshire CCG
03	PC-SafeQuest	12,246	7	5	3	12	2	2	989	Staffordshire CCG
04	PC-SafeQuest	7427	5	3	2	7	1	7	996	Staffordshire Moorlands and Shropshire CCG
05^a^	PC-SafeQuest	6217	6	3	3	6	1	9	974	Staffordshire Moorlands and Shropshire CCG
06^b^	PC-SafeQuest MapSaf	4377	3	1	1	4	1	5	987	Staffordshire CCG
07^a^	PC-SafeQuest MapSaf	9141	7	4	5	8	2	7	964	Staffordshire Moorlands and Shropshire CCG
08	PC-SafeQuest	3919	3	3	0	9	2	6	994	Staffordshire Moorlands and Shropshire CCG
09	PC-SafeQuest MapSaf	11,500	5	4	1	12	3	14	995	Wolverhampton CCG

^a^Dispensing practice, ^b^Teaching practice, ^c^Based on Index of Multiple Deprivation, a compilation of seven domains off poverty including income and health where 1 is the most deprived [21] ^d^QOF maximum score of 1000

**Table 6:** Job role of those interviewed at each practice

	**Practice-01**		**Practice-02**		**Practice-03**		**Practice-04**		**Practice-05**		**Practice-06**		**Practice-07**		**Practice-08**		**Practice-09**		**Total *n***	
	***MS***^**a**^	***SQ***^**a**^	***MS***	***SQ***	***MS***	***SQ***	***MS***	***SQ***	***MS***	***SQ***	***MS***	***SQ***	***MS***	***SQ***	***MS***	***SQ***	***MS***	***SQ***	***MS***	***SQ***
**GP**	2	1		1				1		1	2	2	2	2		1	2	2	8	11
**PM**		1		1		1		1		1			1	1		1	1	1	2	8
**HCA**						1					1	1							1	2
**Pharm.**													1						1	
**P Nurse**	1			1		1					1	1							2	3
**Admin**	3	3		5		3		5		4	4	4	2	2		2	2	2	11	30
***Total***	*6*	*5*		*8*		*6*		*7*		*6*	*8*	*8*	*6*	*5*		*4*	*5*	*5*	*25*	*54*

^a^*MS* MapSaf, *SQ* PC-SafeQuest

##### Qualitative data

The qualitative data is presented below within the domains described previously relevant to early implementation and indicative of longer-term adoption; Acceptability, Appropriateness, and Feasibility [37, 55].

#### Acceptability

Participants described attitudes toward the design and content of both tools and the relative simplicity with which they can be used.

##### PC-SafeQuest

It was felt that the language of the survey questions was straightforward and easy to comprehend, representing concepts that were directly relevant to the systems and processes practices deployed.

*“Well, they were questions that you actually could give an answer to. It was meaningful – you needed to answer, that they were relevant to the surgery, to you in your role, to you and your workmates, you and the practice. It was short, to the point…” Practice-08, Female*

The PC-SafeQuest survey was designed to be completed independently and anonymously and participants appreciated the freedom this granted to answer honestly.

*“I think you get a probably more honest answer when people fill in an anonymised questionnaire in their own time, when they’ve got time to think about it. You’re not pressured by a group environment, by time, by peer pressure.” Practice-08, Male*

Some of the questions in the survey were phrased in the negative to disrupt the “response-order effect” where responders adopt a pattern in answering scaled questions [56]. This did however leave some confused. As one participant at Practice-07 described,

*“Because once or twice I found myself – I knew what the answer I wanted was but then I went back and realised I’d done my scoring the wrong way round, it was completely at the other end!” Practice-07, Female*

##### MaPSaF

Participants felt that prior sight of the tool evaluation sheet that was used to begin the process of group reflection [45] could have helped them shape a more accurate response.

*“Might have been nice to have had this before and actually read it and digested it because it’s a lot to take in.” Practice-01, Female*

This evaluation sheet details a series of patient safety related concepts [45], and some felt the prescriptive nature of the text failed to accurately reflect processes specific to their individual practice. As one participant at Practice-07 explained this left them unsure as to how accurately they had portrayed their patient safety culture.

*“We marked ourselves down on some things because of the wording, like you were saying ‘electronic’, or ‘the patient involvement in the training’ and all that, and I think it’s the wording around those—because it shouldn’t reflect a mark down really for us—should it? Because we’re just doing it in a different format that better suits the practice?” Practice-07, Female*

The MapSaf tool originated in industry [35] and some felt the language and terminology remained redolent of its industrial background and less pertinent to their experience of modern healthcare practice.

*“It feels like it’s come from business and I don’t think it’s made the transition has it from the business world? It does feel like a ‘Shell’ document still” Practice-05, Male*

#### Appropriateness

Participants described the relative success with which the tools produced actionable outputs relevant to the way care was provided at their particular GP practice.

##### PC-SafeQuest

The PC-SafeQuest produced outputs that could inform practice wide discussions. For example a participant at Practice-02 felt the report could provide structure to conversations about the various dimensions of safety.

*“I think it would be quite useful for us… the outcome, you know, what answers came up could be discussed at a practice meeting when everybody’s present, you know? Everybody can have their input and as an add-on to what we would ordinarily do at a practice meeting when we have significant events and all that sort of stuff… I think it would be useful to be used in conjunction with that.” Practice-02, Female*

At Practice 09 one participant felt that the ability of the tool to grant an oversight of different areas of practice operations was a useful attribute, enabling the development of a coherent plan for improving safety involving multiple aspects of service delivery.

*“I think we would have to look at the feedback in different [staff] groups…and so work out ‘how did that happen? Why have we got such different numbers?’ But if actually the practice all came out with very much the same sort of things then you can do a development plan for your whole practice together. So I think it gives you a lot of information…” Practice-09, Female*

##### MaPSaF

MapSaf enabled synchronous and open discussion across staff groups and its ability to create a broader understanding of the experiences of colleagues fulfilling different roles was described.

*“Talking to someone from a different clinical area or organisational area was really important because it just gives you that chance to see that other viewpoint and think—‘actually, yeah we might do it particularly well in our area but the rest of the organisation might be doing it really well!’ and it gives you a chance to do that.” Practice- 01, Male*

Having such cross-practice discussions moderated by a neutral arbitrator that enabled all attendees to have an equal voice was also recognised as a valuable characteristic of the tool.

*“…you need an external facilitator that says – ‘What made you say that?’ and will actually say to somebody ‘You’ve got licence to talk because I'm asking you!’ “ Practice 09, Female*

#### Feasibility

Participants described the practicability of introducing the tool into regular or routine daily use, including the potential long-term benefits and the resources required.

##### PC-SafeQuest

The quantitative output of PC-SafeQuest offered the opportunity for a temporal comparison with previous scores at the same practice. As one participant at Practice-09 opined,

*“It’s good to give you a comparator isn’t it? ‘Where were we last year? Where are we now?’ and ‘Who thinks differently? How is it the clinicians see this different to the non-clinicians? Managers to non-managers?’” Practice-09 Female*

Another strength that was cited as contributing to its continued use was the way its inclusivity (i.e. it was completed by the full range of practice staff) would help embed a safety culture throughout the practice team. As a participant in Practice -01 explained,

*“…by involving everyone, you are just doing it as part of that ‘embedding the culture’ because it’s everyone seeing that actually they're totally involved in it you know? It isn't just a clinician, everyone is doing their bit and their bit is important, so whatever you do to a patient is important and it’s [all] part of it and I think that’s really important, quite powerful actually!” Practice-01, Male*

##### MaPSaF

In considering the sustained uptake of MaPSaF, the benefits of its thorough and detailed approach were recognised by a number of participants.

*“I think it helps you understand ‘what do these things mean?’ Because reading it makes you stop and think about ‘What do I do? How many of these things have I glossed over?’ So just reading in detail… it is learning without even realising you’re learning – so from that point of view I think the practice to be exposed to [it] is good …” Practice-08, Female*

However, another participant sounded a note of caution that though it offered valuable information its complexity would likely prove inhibitive to its future use.

*“… it is probably an extremely good place to start because I think it’s very comprehensive. I think the problem is it’s far too wordy and far too comprehensive and I don’t think it works well in this particular environment.” Practice-07, Male*

## Discussion

### Summary of main findings

Our comparative evaluation of the content and usability of the two tools specifically designed to assess patient safety culture in UK primary care that are supported by the RCGP [32] provides senior staff with the practical insight that enables them to make a more informed decision on which to use and when. It is particularly timely considering the impact on patient safety of the sudden and substantive changes to care and the working environment experienced in general practice [24, 57].

The content analysis revealed that neither tool assessed all ten of the recommended dimensions of safety culture though between them nine were covered with the exception being performance incentives. The qualitative data revealed how both tools were appreciated for their ability to offer practicable insights into patient safety culture and facilitate cross-practice reflection on the safety of the care their practices provided. The quantitative PC-SafeQuest tool was recommended for being straightforward in concept and practice. It could be completed quickly, independently, and remotely whilst still capturing relevant data. However the speed with which this these reports were discussed or acted upon was dependent upon the discretion of senior staff [36]. In contrast the qualitative MaPSaF tool possessed greater immediacy by enabling synchronous in-depth discussion amongst a range of staff; promoting challenge and exploring emergent issues, though it was reliant upon the potentially difficult task of gathering a range of staff at a single time and location for several hours [45].

### Strengths and limitations

This is the first comparative evaluation of two tools specifically developed to explore patient safety culture in the UK primary care and the practices which took part in our study represented a range of patient populations and practice sizes with a broad mix of clinical and non-clinical staff contributing to the focus group discussions. It was decided to retain the anonymity of participants because of concerns about deductive disclosure in view of the comparatively small sample size though we acknowledge that by not including personal characteristics a degree of insight for the reader may be lost [48, 58]. The length of the focus groups might be considered relatively short yet this can be explained by consensual theory whereby the tight focus of the discussion and the experience of those involved reduces the length of the discussion yet still produces valuable data [59]. This data proved capable of fulfilling the dual aims of the work which were to support the development of a coherent patient safety toolkit as well as provide practical information to GPs and other senior practice staff on the utility and usability of both tools. Ultimately only four practices completed the MapSaf tool citing a lack of the necessary time and though this reduced the amount of feedback it did tally with the comments of those that used the tool around the prohibitive length of the workshop.

### Comparison with existing literature

Despite a long-standing and increasing awareness of the importance of patient safety and the tools created in its support [60, 61] the improvements witnessed in other settings had failed to materialise in primary care [62, 63] motivating the development of a comprehensive patient safety toolkit to tackle a range of issues [40]. In its compilation the need to address the importance of safety culture became apparent [30, 64] and two tools were identified to fulfil this requirement, PC-SafeQuest and MaPSaF. Both were specifically developed for use in the UK to enable the cultural underpinning of collaborative safety-conscious care [36, 65] and both promoted the inclusion of the wider practice team in shaping and fulfilling practice objectives, strategies, and processes relating to patient safety [66, 67]. Their differing methods meant it was initially uncertain which tool would be included in the PST; PC-SafeQuest was brief yet offered a useful cross-sectional assessment of safety culture [36] whereas the more resource intensive MaPSaF [35] was better placed to capture safety culture in its broadest form and explore practice specific perspectives [60].

Participants recognised the ability of both tools to promote and inform personal and team-based reflection on patient safety which led ultimately to the inclusion of both in the PST [68]. The anonymity of PC-SafeQuest meant honest opinions were aired and in the case of MaPSaF the use of a ‘neutral’ moderator mitigated imported hierarchies to the extent all felt free to express their opinions [69]. The reflective practice that both tools encouraged, i.e. the application of critical thinking to improve professional performance [70, 71] supports learning from experience, and aids decision-making in complex clinical settings [72, 73]. Both tools also promoted reflexivity, the related process whereby individuals examine how their preconceptions, judgments and actions influenced how they fulfil their particular role [74]. Exercises promoting individual and group reflection have long been used in the education of providers in fields such as social care and psychology [72, 75] and are increasingly included in the curriculum of pharmacists [76] and medical students [77] suggesting that PC-SafeQuest or MaPSaF might also be employed as educational tools or in continuing professional development for primary care staff.

Primary care is increasingly reliant on a range of staff of different backgrounds, qualifications and experiences and both tools aimed to promote cross-practice collaboration in improving safety and their use was predicated on involving a range of clinical and non-clinical staff. They successfully raised the awareness of safety issues faced by colleagues in other areas that might otherwise have remained unidentified and such inter-professional insight has recently been recommended by the European Forum for Primary Care for its ability to improve communication, and a team ethos and ultimately patient care [78, 79].

## Conclusions

At the time of the study pressure on the UK primary care and in particular general practice was already increasing [80] and has since been exacerbated by the redesign of care processes and the working environment as a result of the Covid-19 pandemic [26]. Such mandatory, top-down changes run contrary to the widely understood principles of safe care which are based on broad stakeholder consultation and engagement [81–85] and highlights the importance of a resilient patient safety culture within practice organisations [86]. The tools studied here can gather relevant information on the major domains of patient safety culture in a reliable way through different approaches that allow individual practices to decide which suits them best at a particular point in time, dependent upon their current resources and priorities. It would appear that PC-SafeQuest’s ability to enable a snapshot of safety culture without the logistical difficulties of convening multiple workshops is more suited for bigger practices employing large numbers of staff across multiple sites. By contrast MapSaf might be favoured by smaller practices where a moderated discussion involving the majority of the practice team can grant a more immediate shared understanding of the experiences of a range of staff groups.

## Data Availability

The datasets generated and analysed during the current study are not publicly available due to reason of participant confidentiality as explained in the manuscript but are available from the corresponding author on reasonable request.

## Abbreviations

PC—SafeQuest: Primary Care Safety Questionnaire
MapSaf: Manchester Patient Safety Framework
GP: General Practitioner
PST: Patient Safety Toolkit
UK: United Kingdom
NHS: National Health Service
RCGP: Royal College of General Practitioners
CCG: Clinical Commissioning Group
NIHR: National Institute for Health Research
CRN: Clinical Research Network
